# The dynamic organization of fungal acetyl-CoA carboxylase

**DOI:** 10.1038/ncomms11196

**Published:** 2016-04-13

**Authors:** Moritz Hunkeler, Edward Stuttfeld, Anna Hagmann, Stefan Imseng, Timm Maier

**Affiliations:** 1 Department Biozentrum, University of Basel, Klingelbergstrasse 50/70, 4056 Basel, Switzerland

## Abstract

Acetyl-CoA carboxylases (ACCs) catalyse the committed step in fatty-acid biosynthesis: the ATP-dependent carboxylation of acetyl-CoA to malonyl-CoA. They are important regulatory hubs for metabolic control and relevant drug targets for the treatment of the metabolic syndrome and cancer. Eukaryotic ACCs are single-chain multienzymes characterized by a large, non-catalytic central domain (CD), whose role in ACC regulation remains poorly characterized. Here we report the crystal structure of the yeast ACC CD, revealing a unique four-domain organization. A regulatory loop, which is phosphorylated at the key functional phosphorylation site of fungal ACC, wedges into a crevice between two domains of CD. Combining the yeast CD structure with intermediate and low-resolution data of larger fragments up to intact ACCs provides a comprehensive characterization of the dynamic fungal ACC architecture. In contrast to related carboxylases, large-scale conformational changes are required for substrate turnover, and are mediated by the CD under phosphorylation control.

## Abstract (web summary)

Acetyl-CoA carboxylases are central regulatory hubs of fatty acid metabolism and are important targets for drug development in obesity and cancer. Here, the authors demonstrate that the regulation of these highly dynamic enzymes in fungi is governed by a mechanism based on phosphorylation-dependent conformational variability.

## Graphical abstract

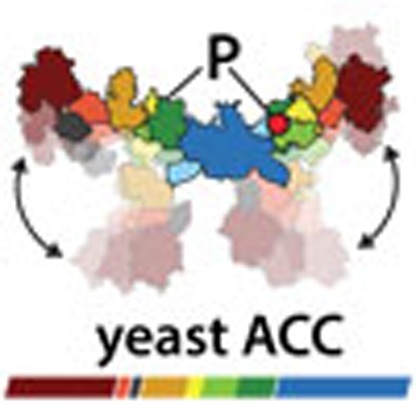

Biotin-dependent acetyl-CoA carboxylases (ACCs) are essential enzymes that catalyse the ATP-dependent carboxylation of acetyl-CoA to malonyl-CoA. This reaction provides the committed activated substrate for the biosynthesis of fatty acids via fatty-acid synthase^1^,^2^. By catalysing this rate-limiting step in fatty-acid biosynthesis, ACC plays a key role in anabolic metabolism. ACC inhibition and knock-out studies show the potential of targeting ACC for treatment of the metabolic syndrome^3^,^4^,^5^. Furthermore, elevated ACC activity is observed in malignant tumours^6^,^7^. A direct link between ACC and cancer is provided by cancer-associated mutations in the breast cancer susceptibility gene 1 (BRCA1), which relieve inhibitory interactions of BRCA1 with ACC^8^,^9^. Thus, ACC is a relevant drug target for type 2 diabetes and cancer^10^,^11^. Microbial ACCs are also the principal target of antifungal and antibiotic compounds, such as Soraphen A^12^,^13^,^14^.

The principal functional protein components of ACCs have been described already in the late 1960s for *Escherichia coli* (*E. coli*) ACC^15^,^16^: Biotin carboxylase (BC) catalyses the ATP-dependent carboxylation of a biotin moiety, which is covalently linked to the biotin carboxyl carrier protein (BCCP). Carboxyltransferase (CT) transfers the activated carboxyl group from carboxybiotin to acetyl-CoA to yield malonyl-CoA. Prokaryotic ACCs are transient assemblies of individual BC, CT and BCCP subunits^17^. Eukaryotic ACCs, instead, are multienzymes, which integrate all functional components into a single polypeptide chain of ∼2,300 amino acids^2^. Human ACC occurs in two closely related isoforms, ACC1 and 2, located in the cytosol and at the outer mitochondrial membrane, respectively^18^,^19^. In addition to the canonical ACC components, eukaryotic ACCs contain two non-catalytic regions, the large central domain (CD) and the BC–CT interaction domain (BT). The CD comprises one-third of the protein and is a unique feature of eukaryotic ACCs without homologues in other proteins. The function of this domain remains poorly characterized, although phosphorylation of several serine residues in the CD regulates ACC activity^20^,^21^,^22^. The BT domain has been visualized in bacterial carboxylases, where it mediates contacts between α- and β-subunits^23^,^24^.

Structural studies on the functional architecture of intact ACCs have been hindered by their huge size and pronounced dynamics, as well as the transient assembly mode of bacterial ACCs. However, crystal structures of individual components or domains from prokaryotic and eukaryotic ACCs, respectively, have been solved^25^,^26^,^27^,^28^,^29^. The structure determination of the holoenzymes of bacterial biotin-dependent carboxylases, which lack the characteristic CD, such as the pyruvate carboxylase (PC)^30^, propionyl-CoA carboxylase^23^, 3-methyl-crotonyl-CoA carboxylase^24^ and a long-chain acyl-CoA carboxylase^31^ revealed strikingly divergent architectures despite a general conservation of all functional components. In these structures, the BC and CT active sites are at distances between 40 and 80 Å, such that substrate transfer could be mediated solely by the mobility of the flexibly tethered BCCP.

Human ACC1 is regulated allosterically, via specific protein–protein interactions, and by reversible phosphorylation. Dynamic polymerization of human ACC1 is linked to increased activity and is regulated allosterically by the activator citrate and the inhibitor palmitate^20^,^21^, or by binding of the small protein MIG-12 (ref. 32). Human ACC1 is further regulated by specific phosphorylation-dependent binding of BRCA1 to Ser1263 in the CD. BRCA1 binds only to the phosphorylated form of ACC1 and prevents ACC activation by phosphatase-mediated dephosphorylation^9^,^33^. Furthermore, phosphorylation by AMP-activated protein kinase (AMPK) and cAMP-dependent protein kinase (PKA) leads to a decrease in ACC1 activity. AMPK phosphorylates ACC1 *in vitro* at Ser80, Ser1201 and Ser1216 and PKA at Ser78 and Ser1201. However, regulatory effects on ACC1 activity are mainly mediated by phosphorylation of Ser80 and Ser1201 (refs 34, 35). Phosphorylated Ser80, which is highly conserved only in higher eukaryotes, presumably binds into the Soraphen A-binding pocket^36^. The regulatory Ser1201 shows only moderate conservation across higher eukaryotes, while the phosphorylated Ser1216 is highly conserved across all eukaryotes. However, no effect of Ser1216 phosphorylation on ACC activity has been reported in higher eukaryotes.

For fungal ACC, neither spontaneous nor inducible polymerization has been detected despite considerable sequence conservation to human ACC1. The BRCA1-interacting phosphoserine position is not conserved in fungal ACC, and no other phospho-dependent protein–protein interactions of fungal ACC have been described. In yeast ACC, phosphorylation sites have been identified at Ser2, Ser735, Ser1148, Ser1157 and Ser1162 (ref. 37). Of these, only Ser1157 is highly conserved in fungal ACC and aligns to Ser1216 in human ACC1. Its phosphorylation by the AMPK homologue SNF1 results in strongly reduced ACC activity^22^,^38^.

Despite the outstanding relevance of ACC in primary metabolism and disease, the dynamic organization and regulation of the giant eukaryotic, and in particular fungal ACC, remain poorly characterized. Here we provide the structure of *Saccharomyces cerevisiae* (*Sce*) ACC CD, intermediate- and low-resolution structures of human (*Hsa*) ACC CD and larger fragments of fungal ACC from *Chaetomium thermophilum* (*Cth*; Fig. 1a). Integrating these data with small-angle X-ray scattering (SAXS) and electron microscopy (EM) observations yield a comprehensive representation of the dynamic structure and regulation of fungal ACC.

## Results

### The organization of the yeast ACC CD

**Figure 1:**
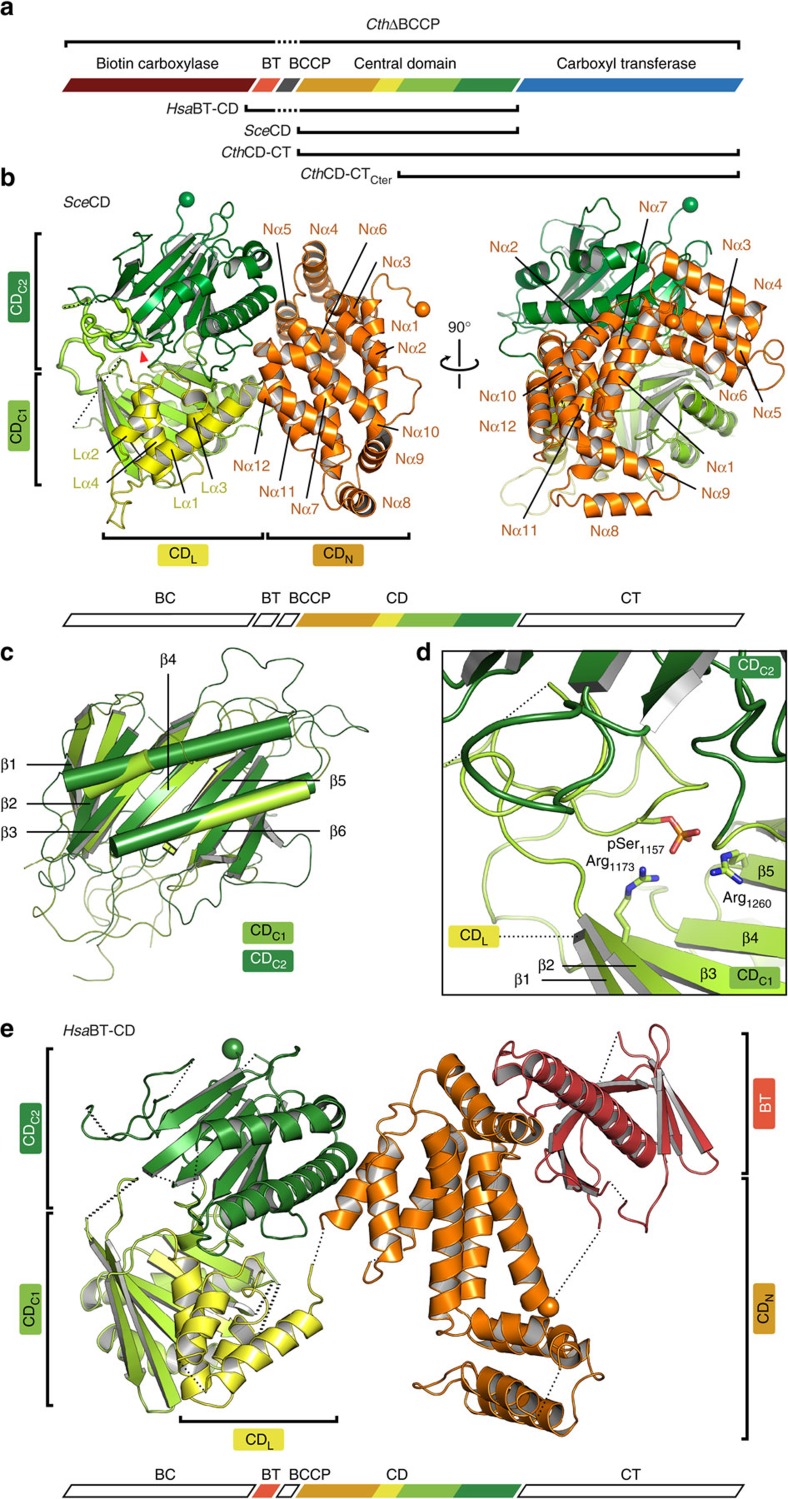
The phosphorylated central domain of yeast ACC. (**a**) Schematic overview of the domain organization of eukaryotic ACCs. Crystallized constructs are indicated. (**b**) Cartoon representation of the *Sce*CD crystal structure. CD_N_ is linked by a four-helix bundle (CD_L_) to two α–β-fold domains (CD_C1_ and CD_C2_). The regulatory loop is shown as bold cartoon, and the phosphorylated Ser1157 is marked by a red triangle. The N- and C termini are indicated by spheres. (**c**) Superposition of CD_C1_ and CD_C2_ reveals highly conserved folds. (**d**) The regulatory loop with the phosphorylated Ser1157 is bound into a crevice between CD_C1_ and CD_C2_, the conserved residues Arg1173 and Arg1260 coordinate the phosphoryl-group. (**e**) Structural overview of *Hsa*BT-CD. The attachment points to the N-terminal BCCP domain and the C-terminal CT domain are indicated with spheres. All colourings are according to scheme **a**.

First, we focused on structure determination of the 82-kDa CD. The crystal structure of the CD of *Sce*ACC (*Sce*CD) was determined at 3.0 Å resolution by experimental phasing and refined to *R*_work_/*R*_free_=0.20/0.24 (Table 1). The overall extent of the *Sce*CD is 70 by 75 Å (Fig. 1b and Supplementary Fig. 1a,b), and the attachment points of the N-terminal 26-residue linker to the BCCP domain and the C-terminal CT domain are separated by 46 Å (the N- and C termini are indicated with spheres in Fig. 1b). *Sce*CD comprises four distinct domains, an N-terminal α-helical domain (CD_N_), and a central four-helix bundle linker domain (CD_L_), followed by two α–β-fold C-terminal domains (CD_C1_/CD_C2_). CD_N_ adopts a letter C shape, where one of the ends is a regular four-helix bundle (Nα3-6), the other end is a helical hairpin (Nα8,9) and the bridging region comprises six helices (Nα1,2,7,10–12). CD_L_ is composed of a small, irregular four-helix bundle (Lα1–4) and tightly interacts with the open face of CD_C1_ via an interface of 1,300 Å^2^ involving helices Lα3 and Lα4. CD_L_ does not interact with CD_N_ apart from the covalent linkage and forms only a small contact to CD_C2_ via a loop between Lα2/α3 and the N-terminal end of Lα1, with an interface area of 400 Å^2^. CD_C1_/CD_C2_ share a common fold; they are composed of six-stranded β-sheets flanked on one side by two long, bent helices inserted between strands β3/β4 and β4/β5. CD_C2_ is extended at its C terminus by an additional β-strand and an irregular β-hairpin. On the basis of a root mean square deviation of main chain atom positions of 2.2 Å, CD_C1_/CD_C2_ are structurally more closely related to each other than to any other protein (Fig. 1c); they may thus have evolved by duplication. Close structural homologues could not be found for the CD_N_ or the CD_C_ domains.

### A regulatory loop mediates interdomain interactions

To define the functional state of insect-cell-expressed ACC variants, we employed mass spectrometry (MS) for phosphorylation site detection. In insect-cell-expressed full-length *Sce*ACC, the highly conserved Ser1157 is the only fully occupied phosphorylation site with functional relevance in *S. cerevisiae*. Additional phosphorylation was detected for Ser2101 and Tyr2179; however, these sites are neither conserved across fungal ACC nor natively phosphorylated in yeast. MS analysis of dissolved crystals confirmed the phosphorylated state of Ser1157 also in *Sce*CD crystals. The *Sce*CD structure thus authentically represents the state of *Sce*ACC, where the enzyme is inhibited by SNF1-dependent phosphorylation.

In the *Sce*CD crystal structure, the phosphorylated Ser1157 resides in a regulatory 36-amino-acid loop between strands β2 and β3 of CD_C1_ (Fig. 1b,d), which contains two additional less-conserved phosphorylation sites (Ser1148 and Ser1162) confirmed in yeast^39^, but not occupied here. This regulatory loop wedges between the CD_C1_ and CD_C2_ domains and provides the largest contribution to the interdomain interface. The N-terminal region of the regulatory loop also directly contacts the C-terminal region of CD_C2_ leading into CT. Phosphoserine 1157 is tightly bound by two highly conserved arginines (Arg1173 and Arg1260) of CD_C1_ (Fig. 1d). Already the binding of phosphorylated Ser1157 apparently stabilizes the regulatory loop conformation; the accessory phosphorylation sites Ser1148 and Ser1162 in the same loop may further modulate the strength of interaction between the regulatory loop and the CD_C1_ and CD_C2_ domains. Phosphorylation of the regulatory loop thus determines interdomain interactions of CD_C1_ and CD_C2_, suggesting that it may exert its regulatory function by modifying the overall structure and dynamics of the CD.

The functional role of Ser1157 was confirmed by an activity assay based on the incorporation of radioactive carbonate into acid non-volatile material^40^. Phosphorylated *Sce*ACC shows only residual activity (*k*_cat_=0.4±0.2 s^−1^, s.d. based on five replicate measurements), which increases 16-fold (*k*_cat_=6.5±0.3 s^−1^) after dephosphorylation with λ protein phosphatase. The values obtained for dephosphorylated *Sce*ACC are comparable to earlier measurements of non-phosphorylated yeast ACC expressed in *E. coli*^41^.

### The variable CD is conserved between yeast and human

To compare the organization of fungal and human ACC CD, we determined the structure of a human ACC1 fragment that comprises the BT and CD domains (*Hsa*BT-CD), but lacks the mobile BCCP in between (Fig. 1a). An experimentally phased map was obtained at 3.7 Å resolution for a cadmium-derivatized crystal and was interpreted by a poly-alanine model (Fig. 1e and Table 1). Each of the four CD domains in *Hsa*BT-CD individually resembles the corresponding *Sce*CD domain; however, human and yeast CDs exhibit distinct overall structures. In agreement with their tight interaction in *Sce*CD, the relative spatial arrangement of CD_L_ and CD_C1_ is preserved in *Hsa*BT-CD, but the human CD_L_/CD_C1_ didomain is tilted by 30° based on a superposition of human and yeast CD_C2_ (Supplementary Fig. 1c). As a result, the N terminus of CD_L_ at helix Lα1, which connects to CD_N_, is shifted by 12 Å. Remarkably, CD_N_ of *Hsa*BT-CD adopts a completely different orientation compared with *Sce*CD. With CD_L_/CD_C1_ superposed, CD_N_ in *Hsa*BT-CD is rotated by 160° around a hinge at the connection of CD_N_/CD_L_ (Supplementary Fig. 1d). This rotation displaces the N terminus of CD_N_ in *Hsa*BT-CD by 51 Å compared with *Sce*CD, resulting in a separation of the attachment points of the N-terminal linker to the BCCP domain and the C-terminal CT domain by 67 Å (the attachment points are indicated with spheres in Fig. 1e). The BT domain of *Hsa*BT-CD consists of a helix that is surrounded at its N terminus by an antiparallel eight-stranded β-barrel. It resembles the BT of propionyl-CoA carboxylase^23^; only the four C-terminal strands of the β-barrel are slightly tilted.

On the basis of MS analysis of insect-cell-expressed human full-length ACC, Ser80 shows the highest degree of phosphorylation (90%). Ser29 and Ser1263, implicated in insulin-dependent phosphorylation and BRCA1 binding, respectively, are phosphorylated at intermediate levels (40%). The highly conserved Ser1216 (corresponding to *S. cerevisiae* Ser1157), as well as Ser1201, both in the regulatory loop discussed above, are not phosphorylated. However, residual phosphorylation levels were detected for Ser1204 (7%) and Ser1218 (7%) in the same loop. MS analysis of the *Hsa*BT-CD crystallization sample reveals partial proteolytic digestion of the regulatory loop. Accordingly, most of this loop is not represented in the *Hsa*BT-CD crystal structure. The absence of the regulatory loop might be linked to the less-restrained interface of CD_L_/CD_C1_ and CD_C2_ and altered relative orientations of these domains. Besides the regulatory loop, also the phosphopeptide target region for BRCA1 interaction is not resolved presumably because of pronounced flexibility.

At the level of isolated yeast and human CD, the structural analysis indicates the presence of at least two hinges, one with large-scale flexibility at the CD_N_/CD_L_ connection, and one with tunable plasticity between CD_L_/CD_C1_ and CD_C2_, plausibly affected by phosphorylation in the regulatory loop region.

### The integration of CD into the fungal ACC multienzyme

**Figure 2:**
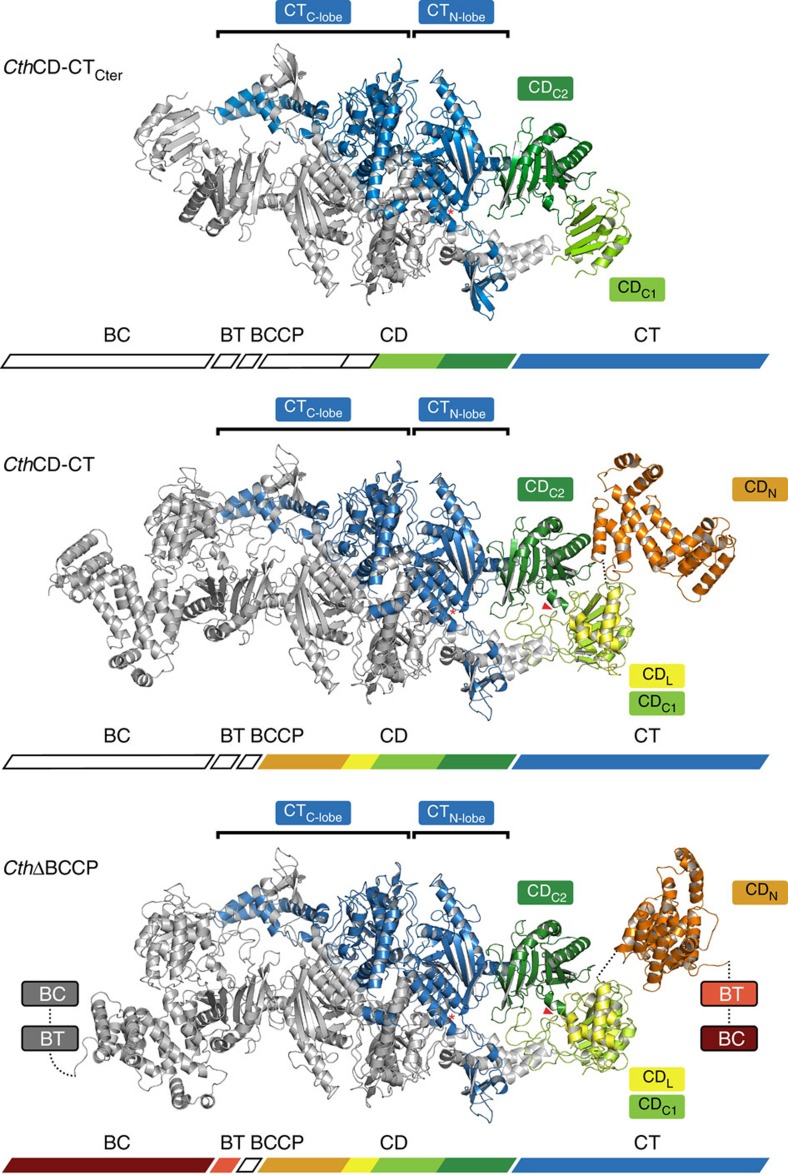
Architecture of the CD–CT core of fungal ACC. Cartoon representation of crystal structures of multidomain constructs of *Cth*ACC. One protomer is shown in colour and one in grey. Individual domains are labelled; the active site of CT and the position of the conserved regulatory phosphoserine site based on *Sce*CD are indicated by an asterisk and a triangle, respectively.

To further obtain insights into the functional architecture of fungal ACC, we characterized larger multidomain fragments up to the intact enzymes. Using molecular replacement based on fungal ACC CD and CT models, we obtained structures of a variant comprising *Cth*CT and CD_C1_/CD_C2_ in two crystal forms at resolutions of 3.6 and 4.5 Å (*Cth*CD-CT_Cter1/2_), respectively, as well as of a *Cth*CT linked to the entire CD at 7.2 Å resolution (*Cth*CD-CT; Figs 1a and 2, Table 1). No crystals diffracting to sufficient resolution were obtained for larger BC-containing fragments, or for full-length *Cth* or *Sce*ACC. To improve crystallizability, we generated ΔBCCP variants of full-length ACC, which, based on SAXS analysis, preserve properties of intact ACC (Supplementary Table 1 and Supplementary Fig. 2a–c). For *Cth*ΔBCCP, crystals diffracting to 8.4 Å resolution were obtained. However, molecular replacement did not reveal a unique positioning of the BC domain. Owing to the limited resolution the discussion of structures of *Cth*CD-CT and *Cth*ΔBCCP is restricted to the analysis of domain localization. Still, these structures contribute considerably to the visualization of an intrinsically dynamic fungal ACC.

**Figure 3:**
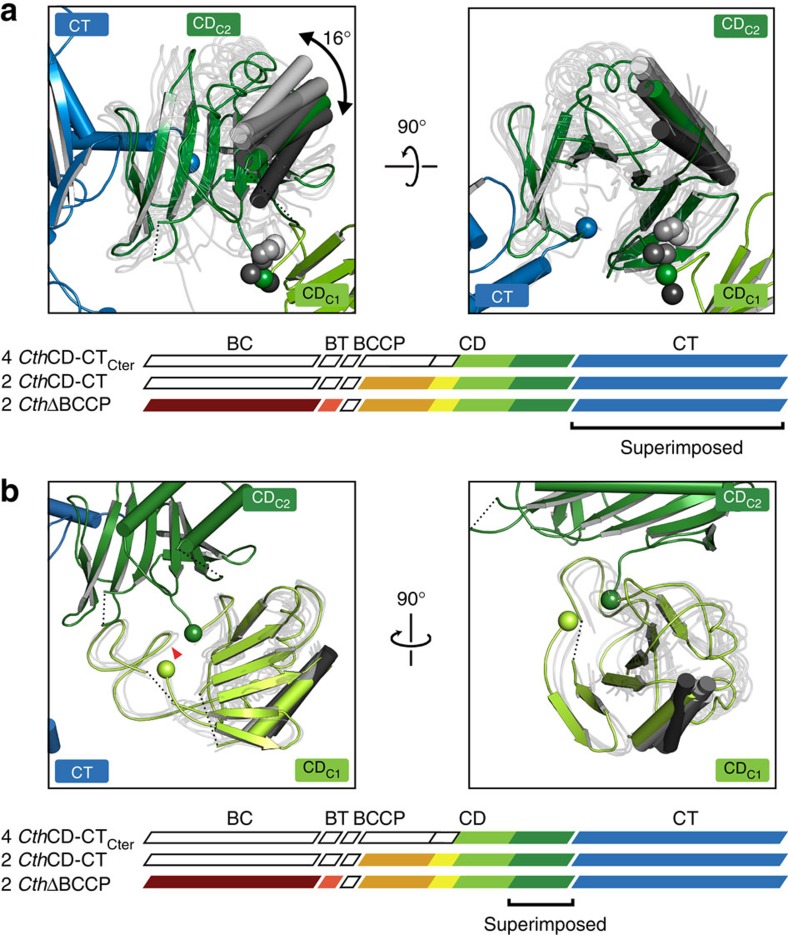
Variability of the connections of CD_C2_ to CT and CD_C1_ in fungal ACC. (**a**) Hinge properties of the CD_C2_–CT connection analysed by a CT-based superposition of eight instances of the CD_C2_-CT segment. For clarity, only one protomer of *Cth*CD-CT_Cter1_ is shown in full colour as reference. For other instances, CD_C2_ domains are shown in transparent tube representation with only one helix each highlighted. The range of hinge bending is indicated and the connection points between CD_C2_ and CT (blue) as well as between CD_C1_ and CD_C2_ (green and grey) are marked as spheres. (**b**) The interdomain interface of CD_C1_ and CD_C2_ exhibits only limited plasticity. Representation as in **a**, but the CD_C1_ and CD_C2_ are superposed based on CD_C2_. One protomer of *Cth*ΔBCCP is shown in colour, the CD_L_ domains are omitted for clarity and the position of the phosphorylated serine based on *Sce*CD is indicated with a red triangle. The connection points from CD_C1_ to CD_C2_ and to CD_L_ are represented by green spheres.

In all these crystal structures, the CT domains build a canonical head-to-tail dimer^29^, with active sites formed by contributions from both protomers (Fig. 2 and Supplementary Fig. 3a). The connection of CD and CT is provided by a 10-residue peptide stretch, which links the N terminus of CT to the irregular β-hairpin/β-strand extension of CD_C2_ (Supplementary Fig. 3b). The connecting region is remarkably similar in isolated CD and *Cth*CD-CT_Cter_ structures, indicating inherent conformational stability. CD/CT contacts are only formed in direct vicinity of the covalent linkage and involve the β-hairpin extension of CD_C2_ as well as the loop between strands β2/β3 of the CT N-lobe, which contains a conserved RxxGxN motif. The neighbouring loop on the CT side (between CT β1/β2) is displaced by 2.5 Å compared to isolated CT structures (Supplementary Fig. 3c). On the basis of an interface area of ∼600 Å^2^ and its edge-to-edge connection characteristics, the interface between CT and CD might be classified as conformationally variable. Indeed, the comparison of the positioning of eight instances of the C-terminal part of CD relative to CT in crystal structures determined here, reveals flexible interdomain linking (Fig. 3a). The CD_C2_/CT interface acts as a true hinge with observed rotation up to 16°, which results in a translocation of the distal end of CD_C2_ by 8 Å.

The interface between CD_C2_ and CD_L_/CD_C1_, which is mediated by the phosphorylated regulatory loop in the *Sce*CD structure, is less variable than the CD–CT junction, and permits only limited rotation and tilting (Fig. 3b). Analysis of the impact of phosphorylation on the interface between CD_C2_ and CD_L_/CD_C1_ in *Cth*ACC variant structures is precluded by the limited crystallographic resolution. However, MS analysis of *Cth*CD-CT and *Cth*ΔBCCP constructs revealed between 60 and 70% phosphorylation of Ser1170 (corresponding to *Sce*ACC Ser1157).

**Figure 4:**
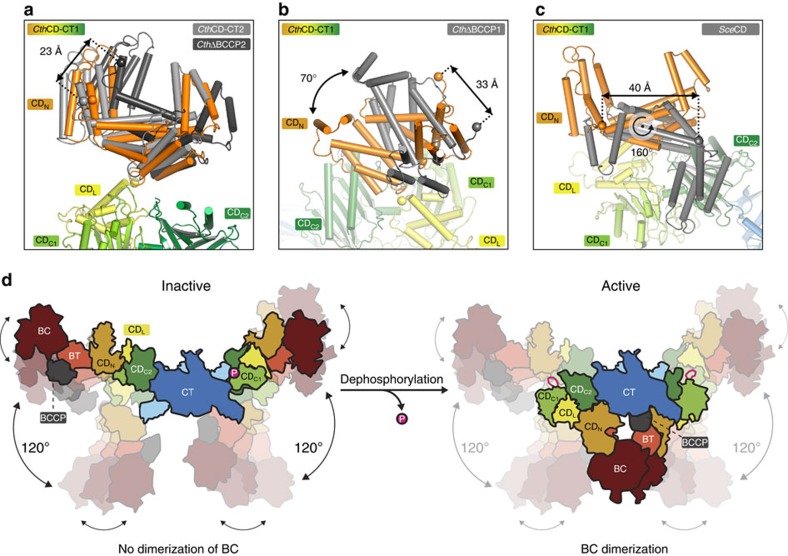
The conformational dynamics of fungal ACC. (**a**–**c**) Large-scale conformational variability of the CD_N_ domain relative to the CD_L_/CD_C1_ domain. *Cth*CD-CT1 (in colour) serves as reference, the compared structures (as indicated, numbers after construct name differentiate between individual protomers) are shown in grey. Domains other than CD_N_ and CD_L_/CD_C1_ are omitted for clarity. The domains are labelled and the distances between the N termini of CD_N_ (spheres) in the compared structures are indicated. (**d**) Schematic model of fungal ACC showing the intrinsic, regulated flexibility of CD in the phosphorylated inhibited or the non-phosphorylated activated state. Flexibility of the CD_C2_/CT and CD_N_/CD_L_ hinges is illustrated by arrows. The Ser1157 phosphorylation site and the regulatory loop are schematically indicated in magenta.

The CD_N_ domain positioning relative to CD_L_/CD_C1_ is highly variable with three main orientations observed in the structures of *Sce*CD and the larger *Cth*ACC fragments: CD_N_ tilts, resulting in a displacement of its N terminus by 23 Å (Fig. 4a, observed in both protomers of *Cth*CD-CT and one protomer of *Cth*ΔBCCP, denoted as *Cth*CD-CT1/2 and *Cth*ΔBCCP1, respectively). In addition, CD_N_ can rotate around hinges in the connection between CD_N_/CD_L_ by 70° (Fig. 4b, observed in the second protomer of *Cth*ΔBCCP, denoted as *Cth*ΔBCCP2) and 160° (Fig. 4c, observed in *Sce*CD) leading to displacement of the anchor site for the BCCP linker by up to 33 and 40 Å, respectively.

Conformational variability in the CD thus contributes considerably to variations in the spacing between the BC and CT domains, and may extend to distance variations beyond the mobility range of the flexibly tethered BCCP. On the basis of the occurrence of related conformational changes between fungal and human ACC fragments, the observed set of conformations may well represent general states present in all eukaryotic ACCs.

### Large-scale conformational variability of fungal ACC

To obtain a comprehensive view of fungal ACC dynamics in solution, we employed SAXS and EM. SAXS analysis of *Cth*ACC agrees with a dimeric state and an elongated shape with a maximum extent of 350 Å (Supplementary Table 1). The smooth appearance of scattering curves and derived distance distributions might indicate substantial interdomain flexibility^42^ (Supplementary Fig. 2a–c). Direct observation of individual full-length C*th*ACC particles, according to MS results predominantly in a phosphorylated low-activity state, in negative stain EM reveals a large set of conformations from rod-like extended to U-shaped particles. Class averages, obtained by maximum-likelihood-based two-dimensional (2D) classification, are focused on the dimeric CT domain and the full BC–BCCP–CD domain of only one protomer, due to the non-coordinated motions of the lateral BC/CD regions relative to the CT dimer. They identify the connections between CD_N_/CD_L_ and between CD_C2_/CT as major contributors to conformational heterogeneity (Supplementary Fig. 4a,b). The flexibility in the CD_C2_/CT hinge appears substantially larger than the variations observed in the set of crystal structures. The BC domain is not completely disordered, but laterally attached to BT/CD_N_ in a generally conserved position, albeit with increased flexibility. Surprisingly, in both the linear and U-shaped conformations, the approximate distances between the BC and CT active sites would remain larger than 110 Å. These observed distances are considerably larger than in static structures of any other related biotin-dependent carboxylase. Furthermore, based on an average length of the BCCP–CD linker in fungal ACC of 26 amino acids, mobility of the BCCP alone would not be sufficient to bridge the active sites of BC and CT. Consequently, increased flexibility or additional modes of conformational changes may be required for productive catalysis. The most relevant candidate site for mediating such additional flexibility and permitting an extended set of conformations is the CD_C1_/CD_C2_ interface, which is rigidified by the Ser1157-phosphorylated regulatory loop, as depicted in the *Sce*CD crystal structure.

## Discussion

Altogether, the architecture of fungal ACC is based on the central dimeric CT domain (Fig. 4d). The CD consists of four distinct subdomains and acts as a tether from the CT to the mobile BCCP and an oriented BC domain. The CD has no direct role in substrate recognition or catalysis but contributes to the regulation of all eukaryotic ACCs. In higher eukaryotic ACCs, regulation via phosphorylation is achieved by combining the effects of phosphorylation at Ser80, Ser1201 and Ser1263. In fungal ACC, however, Ser1157 in the regulatory loop of the CD is the only phosphorylation site that has been demonstrated to be both phosphorylated *in vivo* and involved in the regulation of ACC activity. In its phosphorylated state, the regulatory loop containing Ser1157 wedges between CD_C1_/CD_C2_ and presumably limits the conformational freedom at this interdomain interface. However, flexibility at this hinge may be required for full ACC activity, as the distances between the BCCP anchor points and the active sites of BC and CT observed here are such large that mobility of the BCCP alone is not sufficient for substrate transfer. The current data thus suggest that regulation of fungal ACC is mediated by controlling the dynamics of the unique CD, rather than directly affecting catalytic turnover at the active sites of BC and CT. A comparison between fungal and human ACC will help to further discriminate mechanistic differences that contribute to the extended control and polymerization of human ACC.

Most recently, a crystal structure of near full-length non-phosphorylated ACC from *S. cerevisae* (lacking only 21 N-terminal amino acids, here denoted as flACC) was published by Wei and Tong^41^. In flACC, the ACC dimer obeys twofold symmetry and assembles in a triangular architecture with dimeric BC domains (Supplementary Fig. 5a). In their study, mutational data indicate a requirement for BC dimerization for catalytic activity. The transition from the elongated open shape, observed in our experiments, towards a compact triangular shape is based on an intricate interplay of several hinge-bending motions in the CD (Fig. 4d). Comparison of flACC with our *Cth*ΔBCCP structure reveals the CD_C2_/CT hinge as a major contributor to conformational flexibility (Supplementary Fig. 5b,c). In flACC, CD_C2_ rotates ∼120° with respect to the CT domain. A second hinge can be identified between CD_C1_/CD_C2_. On the basis of a superposition of CD_C2_, CD_C1_ of the phosphorylated *Sce*CD is rotated by 30° relative to CD_C1_ of the non-phosphorylated flACC (Supplementary Fig. 5d), similar to what we have observed for the non-phosphorylated *Hsa*BT-CD (Supplementary Fig. 1d). When inspecting all individual protomer and fragment structures in their study, Wei and Tong also identify the CD_N_/CD_C1_ connection as a highly flexible hinge, in agreement with our observations.

The only bona fide regulatory phophorylation site of fungal ACC in the regulatory loop is directly participating in CD_C1_/CD_C2_ domain interactions and thus stabilizes the hinge conformation. In flACC, the regulatory loop is mostly disordered, illustrating the increased flexibility due to the absence of the phosphoryl group. Only in three out of eight observed protomers a short peptide stretch (including Ser1157) was modelled. In those instances the Ser1157 residue is located at a distance of 14–20 Å away from the location of the phosphorylated serine observed here, based on superposition of either CD_C1_ or CD_C2_. Applying the conformation of the CD_C1_/CD_C2_ hinge observed in *Sce*CD on flACC leads to CD_N_ sterically clashing with CD_C2_ and BT/CD_N_ clashing with CT (Supplementary Fig. 6a,b). Thus, in accordance with the results presented here, phosphorylation of Ser1157 in *Sce*ACC most likely limits flexibility in the CD_C1_/CD_C2_ hinge such that activation through BC dimerization is not possible (Fig. 4d), which however does not exclude intermolecular dimerization. In addition, EM micrographs of phosphorylated and dephosphorylated *Sce*ACC display for both samples mainly elongated and U-shaped conformations and reveal no apparent differences in particle shape distributions (Supplementary Fig. 7). This implicates that the triangular shape with dimeric BC domains has a low population also in the active form, even though a biasing influence of grid preparation cannot be excluded completely.

Large-scale conformational variability has also been observed in most other carrier protein-based multienzymes, including polyketide^43^ and fatty-acid synthases (with the exception of fungal-type fatty-acid synthases)^44^,^45^, non-ribosomal peptide synthetases^46^ and the pyruvate dehydrogenase complexes^47^, although based on completely different architectures. Together, this structural information suggests that variable carrier protein tethering is not sufficient for efficient substrate transfer and catalysis in any of these systems. The determination of a set of crystal structures of *Sce*ACC in two states, unphosphorylated^41^ and phosphorylated at the major regulatory site Ser1157, provides a unique depiction of multienzyme regulation by post-translational modification (Fig. 4d). The phosphorylated regulatory loop binds to an allosteric site at the interface of two non-catalytic domains and restricts conformational freedom at several hinges in the dynamic ACC. It disfavours the adoption of a rare, compact conformation, in which intramolecular dimerization of the BC domains^41^ results in catalytic turnover. The regulation of activity thus results from restrained large-scale conformational dynamics rather than a direct or indirect influence on active site structure. To our best knowledge, ACC is the first multienzyme for which such a phosphorylation-dependent mechanical control mechanism has been visualized. However, the example of ACC now demonstrates the possibility of regulating activity by controlled dynamics of non-enzymatic linker regions also in other families of carrier-dependent multienzymes. Understanding such structural and dynamic constraints imposed by scaffolding and linking in carrier protein-based multienzyme systems is a critical prerequisite for engineering of efficient biosynthetic assembly lines.

## Methods

### Protein expression and purification

All proteins were expressed in the Baculovirus Expression Vector System. The MultiBac insect cell expression plasmid pACEBACI (Geneva Biotech) was modified to host a GATEWAY (LifeTechnologies) cassette with an N-terminal 10xHis-tag, named pAB1GW-NH10 hereafter. Full-length *Hsa*ACC (Genebank accession #Q13085), *Sce*ACC (#Q00955) and *Cth*ACC (#G0S3L5) were cloned into pAB1GW-NH10 using GATEWAY according to the manufacturer's manual. Truncated variants were constructed by PCR amplification, digestion of the template DNA with DpnI, phosphorylation of the PCR product and religation of the linear fragment to a circular plasmid. The following constructs were used for this study: *Sce*ACC (1–2,233), *Cth*ACC (1–2,297), *Cth*ΔBCCP (1–2,297, Δ700–765), *Cth*CD-CT (788–2,297), *Cth*CD-CT_Cter_ (1,114–2,297), *Sce*CD (768–1,494) and *Hsa*BT-CD (622–1,584, Δ753–818). Bacmid and virus production was carried out according to MultiBac instructions^48^. Baculovirus generation and amplification as well as protein expression were performed in Sf21 cells (Expression Systems) in Insect-Xpress medium (Lonza). The cells were harvested 68–96 h post infection by centrifugation and stored at −80 °C until being processed.

Cells were lysed by sonication and the lysate was cleared by ultracentrifugation. Soluble protein was purified using Ni-NTA (Genscript) and size exclusion chromatography (Superose 6, GE Healthcare). The affinity tag was removed by tobacco etch virus (TEV) protease cleavage overnight at 4 °C. TEV protease and uncleaved protein were removed by orthogonal Ni-NTA purification before size exclusion chromatography. *Sce*ACC, *Cth*ACC and *Cth*ΔBCCP were further purified by high-resolution anion exchange chromatography before size exclusion chromatography. Purified *Sce*CD, *Cth*CD-CT_Cter_, *Cth*CD-CT, *Cth*ΔBCCP, *Cth*ACC and *Sce*ACC were concentrated to 10 mg ml^−1^ in 30 mM 3-(N-morpholino) propanesulfonic acid (MOPS) pH 7, 200 mM ammonium sulfate, 5% glycerol and 10 mM dithiothreitol. Purified *Hsa*BT-CD was concentrated to 20 mg ml^−1^ in 20 mM bicine pH 8.0, 200 mM NaCl, 5% glycerol and 5 mM tris(2-carboxyethyl) phosphine (TCEP). Proteins were used directly or were stored at −80 °C after flash-freezing in liquid nitrogen.

### Protein crystallization

All crystallization experiments were conducted using sitting drop vapour diffusion. *Sce*CD crystals were grown at 19 °C by mixing protein and reservoir solution (0.1 M BisTrisPropane pH 6.5, 0.05–0.2 M di-sodium malonate, 20–30% polyethylene glycol (PEG) 3350, 10 mM trimethylamine or 2% benzamidine) in a 1:1 or 2:1 ratio. Crystals appeared after several days and continued to grow for 20–200 days. Crystals were cryoprotected by short incubation in mother liquor supplemented with 22% ethylene glycol and flash-cooled in liquid nitrogen. For heavy metal derivatization the crystals were incubated in stabilization solution supplemented with 1 mM Thimerosal or 10 mM EuCl_2_, and then backsoaked for 15 s in stabilization solution without heavy metal.

Initial crystals of *Hsa*BT-CD grew in 0.1 M Tris pH 8.5, 0.35 M tri-potassium citrate and 2–3.5% PEG10000 at 19 °C. After several rounds of optimization, good-quality diffraction crystals were obtained at 19 °C in 0.1 M MES pH 6, 0.25–0.35 M tri-potassium citrate, 2–5% PEG10000 and 0.01–0.04 M cadmium chloride. The protein drop contained a 1:1 ratio of protein and reservoir solution. Crystals grew immediately and stopped growing after 3 days. They were dehydrated and cryoprotected in several steps in artificial mother liquor containing incrementally increasing concentrations of tri-potassium citrate, PEG10000 and ethylene glycol and then flash-cooled in liquid nitrogen. The final solution was composed of 0.1 M MES pH 6, 0.5 M tri-potassium citrate, 6.75% PEG10000, 0.01 M cadmium chloride and 22% ethylene glycol.

*Cth*CD-CT_Cter_ crystals were grown at 19 °C by mixing protein and reservoir solution (0.1 M HEPES pH 7.5, 2–7% Tacsimate pH 7, 7.5–15% PEG monomethyl ether 5000) in a 1:1 ratio. Crystals appeared after several days and continued to grow for up to 2 weeks. Crystals were cryoprotected by short incubation in mother liquor supplemented with 22% ethylene glycol.

*Cth*CD-CT ACC crystals were grown at 19 °C by mixing protein and reservoir solution (0.1 M Bicine pH 8.5–9.5, 4–8% PEG8000) in a 1:1 or 1:2 ratio. Crystals grew 8– 10 days and were cryoprotected by short incubation in mother liquor supplemented with 22% ethylene glycol before flash-cooling in liquid nitrogen.

*Cth*ΔBCCP ACC crystals were grown at 19 °C by mixing protein and reservoir solution (0.1 M Morpheus buffer 3 (Molecular Dimensions, MD2-100-102), 7–12% Morpheus ethylene glycols mix (MD2-100-74), 8–12% PEG4000, 17–23% glycerol) in a 1:1 or 1:2 ratio. Crystals grew up to 3 weeks and were cryoprotected in reservoir solution before flash-cooling in liquid nitrogen.

### Structure determination and analysis of phosphorylation

All X-ray diffraction data were collected at beamlines X06SA (PXI) or X06DA (PXIII) at the Swiss Light Source (SLS, Paul Scherrer Institute, Villigen, Switzerland) equipped with PILATUS detectors. The wavelength of data collection was 1.000 Å for native crystals, and 1.527 and 1.907 Å for crystals derivatized with europium and cadmium, respectively. Raw data were processed using XDS^49^. Molecular replacement was carried out using Phaser 2.5.7 and 2.6.0, density modification was performed using Parrot^50^,^51^ and resolve, multicrystal averaging^52^ was carried out using phenix. All model building procedures were conducted using *Coot*^53^ and figures were prepared using PyMOL (Schrödinger LLC).

Diffraction of initial *Sce*CD crystals in space group P4_3_2_1_2 with unit cell dimensions of *a*=*b*=110.3 Å and *c*=131.7 Å was limited to 3.5 Å. The resolution was improved to 3 Å by addition of trimethylamine or benzamidine to the reservoir solution without significant changes in unit cell dimensions. Crystals derivatized with thimerosal and europium were used for initial SAD phase determination using the SHELXC/D package^54^. Two mercury and four europium sites were located, and an initial model was placed in the resulting maps. Since crystals derivatized with europium were slightly non-isomorphous with a *c* axis length of 127 Å, multicrystal averaging was used for density modification and provided directly interpretable maps. Iterative cycles of model building and refinement in Buster (version 2.10.2; Global Phasing Ltd) converged at *R*_work_/*R*_free_ of 0.20/0.24. The final model lacks the disordered N terminus (amino acids 768–789), an extended loop in the CD_C1_ domain (1,203–1,215), a short stretch (1,147–1,149) preceding the regulatory loop and the two very C-terminal residues (1,493–1,494). On the basis of temperature factor analysis, the start and end of the regulatory loop show higher disorder than the region around the interacting phosphoserine 1157. MS analysis of dissolved crystals detected quantitative phosphorylation of the regulatory Ser1157, as also found for full-length *Sce*ACC, and additionally albeit with much lower occurrence, phosphorylation of Ser790, Ser1137, Ser1148 and Ser1159. A modelled phosphoryl position for Ser1159 could overlap with the one of Ser1157, and might be represented in the crystal. For all other phosphorylation sites no difference density could be observed, probably because of very low occupancy. PDBeFold^55^ was used to search for structural homologues. The thresholds for lowest acceptable percentage of matched secondary structure elements were 70% for the search query and 20% for the result.

Initial *Hsa*BT-CD crystals were obtained in space group I4_1_22 with *a*=*b*=240.1 Å and *c*=768.9 Å and diffracted to 7.5 Å. Optimized and dehydrated crystals also belonged to space group I4_1_22 but with unit cell parameters *a*=*b*=267.3 Å and *c*=210.6 Å and diffracted to a resolution of 3.7 Å. Phase information was obtained from SAD based on bound cadmium ions from the crystallization condition. Six cadmium positions were located in a 4.0-Å resolution data set at 1.9 Å wavelength using SHELXC/D^54^ via the HKL2MAP interface^56^. Density modification and phasing based on this anomalous data set, a 3.7-Å resolution data set at 1.0 Å wavelength and additional non-isomorphous lower-resolution data sets led to a high-quality electron density map. At the intermediate resolution obtained, the map was interpreted by a poly-alanine model, which was guided by predicted secondary structure as well as sequence and structural alignment with *Sce*CD. The final model contains five cadmium ions and refines using phenix^52^ against experimental data with *R*_work_/*R*_free_ of 0.35/0.38, as expected for a poly-alanine model. Two *Hsa*BT-CD monomers are packed in the asymmetric unit via the CD_N_ and BT domains. Density on top of the β-barrel of one BT most likely representing parts of the BT–CD linker guided the assignment of this BT to its linked CD partner domain. This BT-to-CD assignment was further supported by the analysis of an additional lower-resolution crystal form. Cadmium ions were found to participate in crystal packing.

In *Hsa*ACC, phosphorylation at regulatory sites was detected as provided in the main text. No phosphorylation was detected for other phosphosites previously identified in large-scale phosphoproteomics studies, namely serines 5, 23, 25, 48, 53, 78, 488, 786, 1273 (refs 57, 58, 59).

Two different crystal forms were obtained for *Cth*CD-CT_Cter_ (denoted as *Cth*CD-CT_Cter1_ and *Cth*CD-CT_Cter2_), diffracting to 3.6 and 4.5 Å. Both forms packed in space group P2_1_2_1_2_1_ with unit cell constants of *a*=97.7 Å, *b*=165.3 Å and *c*=219.2 Å or *a*=100.2 Å, *b*=153.5 Å and *c*=249.2 Å, respectively. Phases were determined by molecular replacement using a homology model based on *Sce*CT (pdb 1od2) as search model in Phaser^51^,^60^,^61^; multicrystal averaging was applied in density modification. The CT domain was rebuilt and an initial homology model based on the *Sce*CD structure was fitted into difference density for *Cth*CD-CT_Cter1_. Iterative cycles of rebuilding and refinement in Buster converged at *R*_work_/*R*_free_ of 0.20/0.24. The refined CD fragment served as a starting model for rebuilding *Cth*CD-CT_Cter2_ at lower resolution. Coordinate refinement in Buster was additionally guided by reference model restraints and converged at *R*_work_/*R*_free_ of 0.24/0.24. Residues 1,114–1,185, 1,213–1,252, 1,380–1,385, 1,465–1,468 and 2,188–2,195 were disordered in both crystal forms and are not included in the models. Helical regions C terminal to Glu2264 of both protomers of *Cth*CD-CT_Cter1_ and C terminal to Leu2259 and Arg2261 of the two protomers of *Cth*CD-CT_Cter2_, respectively, could not be built unambiguously and were therefore interpreted by placing poly-alanine stretches. Conservation was mapped on the *Cth*CD-CT_Cter1_ crystal structure using al2co^62^ based on a sequence alignment of 367 fungal ACC sequences calculated by Clustal Omega^63^. MS analysis of purified protein detected 7% phosphorylation at Ser1170 (corresponding to Ser1157 in *Sce*CD).

*Cth*CD-CT crystallized in space group P3_1_2_1_2 with unit cell constants of *a*=*b*=195.0 Å and *c*=189.5 Å and crystals diffracted to a resolution of 7.2 Å. The structure was solved by molecular replacement using a model composed of *Cth*CT and CD_C2_ as search model in Phaser. CD_C1_ and CD_N_ were placed manually into the resulting maps, and the model was refined using rigid-body, domain-wise TLS and B-factor refinement and NCS- and reference model-restrained coordinate refinement in Buster to *R*_work_/*R*_free_ of 0.23/0.25. Owing to the low resolution, the maximum allowed B-factor in Buster refinement was increased from the default value of 300–500 Å^2^, minimizing B-factor clipping to 5% of all atoms. Residues 1,033–1,035, 1,134–1,152, 1,213–1,252, 1,380–1,385, 1,465–1,468 and 2,188–2,195 were not included in the models. Helical regions C terminal to Leu2259 and Arg2261 on the two protomers, respectively, were interpreted as described for *Cth*CD-CT_Cter_. Loop conformations, including the regulatory loop, were modelled as observed in *Sce*CD. MS analysis of purified protein detected 60% phosphorylation at Ser1170 (corresponding to Ser1157 in *Sce*CD). Conservation was mapped on the *Cth*CD-CT crystal structure as for *Cth*CD-CT_Cter_.

*Cth*ΔBCCP ACC crystallized in space group P6_4_22 with unit cell constants of *a*=*b*=462.2 Å and *c*=204.6 Å, resolution was limited to 8.4 Å. Structure determination and refinement was performed as for *Cth*CD-CT, with a maximum allowed B-factor of 500 Å^2^, minimizing B-factor clipping to 3% of all atoms. Although substantial difference density is observed, no defined positions of the BT and BC domains could be derived because of disorder or partial *in situ* proteolysis or combinations thereof. In addition, residues 1,032–1,039, 1,134–1,152, 1,213–1,252, 1,380–1,385, 1,465–1,468 and 2,188–2,195 were not included in the model. The MissingAtom macro implemented in Buster was employed to account for missing atoms, the final *R*_work_/*R*_free_ were 0.30/0.32. A region C terminal to Leu2259 on one protomer was interpreted as poly-alanine. Loop conformations, including the regulatory loop, were modelled as observed in *Sce*CD. MS analysis of purified protein detected 70% phosphorylation at Ser1170 (corresponding to Ser1157 in *Sce*CD).

### Small-angle X-ray scattering

Proteins were thawed on ice and dialysed overnight against 30 mM MOPS pH 7, 200 mM ammonium sulfate, 5% glycerol and 10 mM dithiothreitol. Raw scattering data were measured at SAXS beamline B21 at Diamond Light Source. The samples were measured at concentrations of 2.5, 5 and 10 mg ml^−1^. Data were processed using the ATSAS package^64^ according to standard procedures^65^,^66^. A slight increase in scattering in the very low-resolution range was observed with increasing protein concentrations, which may be because of interparticle attraction or minor aggregation. Scattering intensities were thus extrapolated to zero concentration using point-wise extrapolation implemented in Primus^67^. Direct comparison of raw scattering curves demonstrates the similarity of *Cth*ACC and *Cth*ΔBCCP, and the derived values such as Rg and Porod Volume match within expected error margins. Molecular mass estimations based on the SAXS–MOW method^68^ derive values of 534.7 and 534.0 kDa for *Cth*ACC and *Cth*ΔBCCP, respectively. The relative discrepancies to the theoretical weights of 516.8 kDa (*Cth*ACC) and 503.0 kDa (*Cth*ΔBCCP) are 3.5% and 6.2%, respectively, which is in a typical range for this method^68^.

### Electron microscopy

Full-length *Cth*ACC was diluted to 0.01 mg ml^−1^ in 30 mM MOPS pH 7.0, 200 mM ammonium sulfate, 5% glycerol and 10 mM dithiothreitol. Protein sample was adsorbed to a 200-μm copper grid and stained with 2% uranyl acetate. Grids of *Cth*ACC were imaged on a CM-200 microscope (Philips) equipped with a TVIPS F416 4k CMOS camera (Tietz Video and Image Processing Systems). The voltage used was 200 kV, and a magnification of × 50,000 results in a pixel size of 2.14 Å. Initial image processing and particle picking was carried out using Xmipp^69^,^70^. Overall, 22,309 particles were picked semi-automatically from 236 micrographs with a box size of 300 × 300 pixels. After extraction, particles with a *z*-score of more than three were discarded and 22,257 particles were aligned and classified into 48 2D class averages using the maximum-likelihood target function in Fourier space (MLF2D). After 72 iterations, 4,226 additional particles were discarded and the remaining 18,031 particles were re-aligned and classified into 36 classes using MLF2D with a high-resolution cutoff of 30 Å. After 44 iterations the alignment converged and class averages were extracted.

### *In vitro* biotinylation and activity assay

To ensure full functionality, *Sce*ACC was biotinylated *in vitro* using the *E. coli* biotin ligase BirA. The reaction mixture contained 10 μM ACC, 3.7 μM BirA, 50 mM Tris-HCl, pH 8, 5.5 mM MgCl_2_, 0.5 mM biotin, 60 mM NaCl, 3 mM ATP and 10% glycerol, and the reaction was allowed to proceed for 7 h at 30 °C.

The catalytic activity of phosphorylated and dephosphorylated *Sce*ACC was measured by following the incorporation of radioactive ^14^C into acid-stable non-volatile material^40^. Dephosphorylated ACC was prepared by overnight treatment with λ protein phosphatase (New England Biolabs) of partially purified ACC before the final gel filtration step. The removal of the phosphoryl group from Ser1157 was confirmed by MS. The reaction mixture contained 0.5 μg recombinant ACC in 100 mM potassium phosphate, pH 8, 3 mM ATP, 5 mM MgCl_2_, 50 mM NaH^14^CO_3_ (specific activity 7.4 MBq mmol^−1^) and 1 mM acetyl-CoA in a total reaction volume of 100 μl. The reaction mixture was incubated for 15 min at 30 °C, stopped by addition of 200 μl 6 M HCl and subsequently evaporated to dryness at 85 °C. The non-volatile residue was redissolved in 100 μl of water, 1 ml Ultima Gold XR scintillation medium (Perkin Elmer) was added and the ^14^C radioactivity was measured in a Packard Tricarb 2000CA liquid scintillation analyser. Measurements were carried out in five replicates and catalytic activities were calculated using a standard curve derived from measurements of varying concentrations of NaH^14^CO_3_ in reaction buffer.

## Additional information

**Accession codes**: Atomic coordinates and structure factors have been deposited in the Protein Data Bank with accession codes 5I6E (*Sce*CD), 5I87 (*Hsa*BT-CD), 5I6F/5I6G (*Cth*CD-CTCter1/2), 5I6H (*Cth*CD-CT) and 5I6I (*Cth*ΔBCCP).

**How to cite this article**: Hunkeler, M. *et al*. The dynamic organization of fungal acetyl-CoA carboxylase. *Nat. Commun.* 7:11196 doi: 10.1038/ncomms11196 (2016).

## Supplementary Material

Supplementary Information Supplementary Figures 1-7 and Supplementary Table 1

## Figures and Tables

**Table 1:** Crystallographic data collection and refinement statistics.

	***Sce*****CD**	***Sce*****CD Thimerosal**	***Sce*****CD Eu**	***Hsa*****BT-CD**	***Hsa*****BT-CD Cd**^**2+**^	***Cth*****CD-CT**_**Cter1**_	***Cth*****CD-CT**_**Cter2**_	***Cth*****CD-CT**	***Cth***Δ**BCCP**
*Data collection*									
Space group	P4_3_2_1_2	P4_3_2_1_2	P4_3_2_1_2	I4_1_22	I4_1_22	P2_1_2_1_2_1_	P2_1_2_1_2_1_	P3_1_2_1_2	P6_4_22
Cell dimensions									
*a, b, c* (Å)	110.86, 110.86, 131.12	111.22, 111.22, 131.49	108.65, 108.65, 127.36	267.27, 267.27, 210.61	267.67, 267.67, 210.46	97.66, 165.34, 219.23	100.17, 153.45, 249,24	295.02, 295.02, 189.52	462.20, 462.20, 204.64
α, β, γ (°)	90, 90, 90	90, 90, 90	90, 90, 90	90, 90, 90	90, 90, 90	90, 90, 90	90, 90, 90	90, 90, 120	90, 90, 120
Resolution* (Å)	3.0	3.4	4.0	3.7	4.1	3.6	4.5	7.2	8.4
*R*_Merge_†	18.2 (389.6)	20.5 (306.1)	40.6 (327.0)	7.5 (400.9)	15 (730.5)	14.5 (384.5)	27.4 (225.6)	5.6 (302.6)	29.4 (381.7)
CC ½*†	100 (58.3)	99.9 (42.6)	99.9 (48.5)	100 (59.4)	99.8 (73.2)	99.9 (50.9)	99.5 (46.7)	100 (33.3)	99.7 (35)
*I*/*σI*†	24.68 (1.46)	7.99 (0.89)	17.92 (1.85)	21.24 (1.07)	16.53 (1.41)	10.61 (0.97)	6.35 (1.00)	18.95 (0.92)	9.05 (0.9)
Completeness†	99.9 (99.9)	99.6 (100)	99.7 (96.8)	99.8 (99.1)	99.8 (99.7)	99.7 (99.9)	99.4 (98.6)	99.6 (100)	99.1 (99.9)
Redundancy†	39.1 (39.8)	12.1 (14.3)	81.6 (65.2)	13.7 (13.7)	20.9 (19.1)	12.7 (13.5)	6.1 (6.5)	9.9 (10.4)	18.5 (18.2)
									
*Refinement*									
Resolution* (Å)	46.4–3.0			84.5–3.7		49.2–3.6	49.1–4.5	49.9–7.2	50.0–8.4
Reflections	16,928	—	—	40,647	—	41,799	23,340	14,046	12,111
*R*_work_/*R*_free_	0.20/0.24	—	—	0.35/0.38‡	—	0.20/0.24	0.24/0.24	0.23/0.25	0.30/0.32
Number of atoms									
Protein	5,465			6,925		16,592	16,405	22,543	22,445
Waters	43	—	—	—	—	—	—	—	—
Ligand/ion	7	—	—	5	—	—	—	—	—
*B*-factors									
Protein	130			158		226	275	272	250
Waters	84	—	—	—	—	—	—	—	—
Ligand/ion	90	—	—	189	—	—	—	—	—
R.m.s.d.									
RMS (angles, °)	0.97	—	—	0.83	—	1.07	1.11	1.15	1.01
RMS (bonds, Å)	0.01	—	—	0.01	—	0.01	0.01	0.01	0.01

^*^Resolution cutoffs determined based on internal correlation significant at the 0.1% level as calculated by XDS.

^†^Highest-resolution shell is shown in parentheses.

^‡^Modelled only as poly-alanine.
