# Supplementary material for: The dynamic organization of fungal acetyl-CoA carboxylase
Source: Nat Commun. 2016 Apr 13;7:11196. doi: 10.1038/ncomms11196 (PMC4833862; doi:10.1038/ncomms11196)
Supplement: Supplementary Information — Supplementary Figures 1-7 and Supplementary Table 1 [file ncomms11196-s1.pdf]

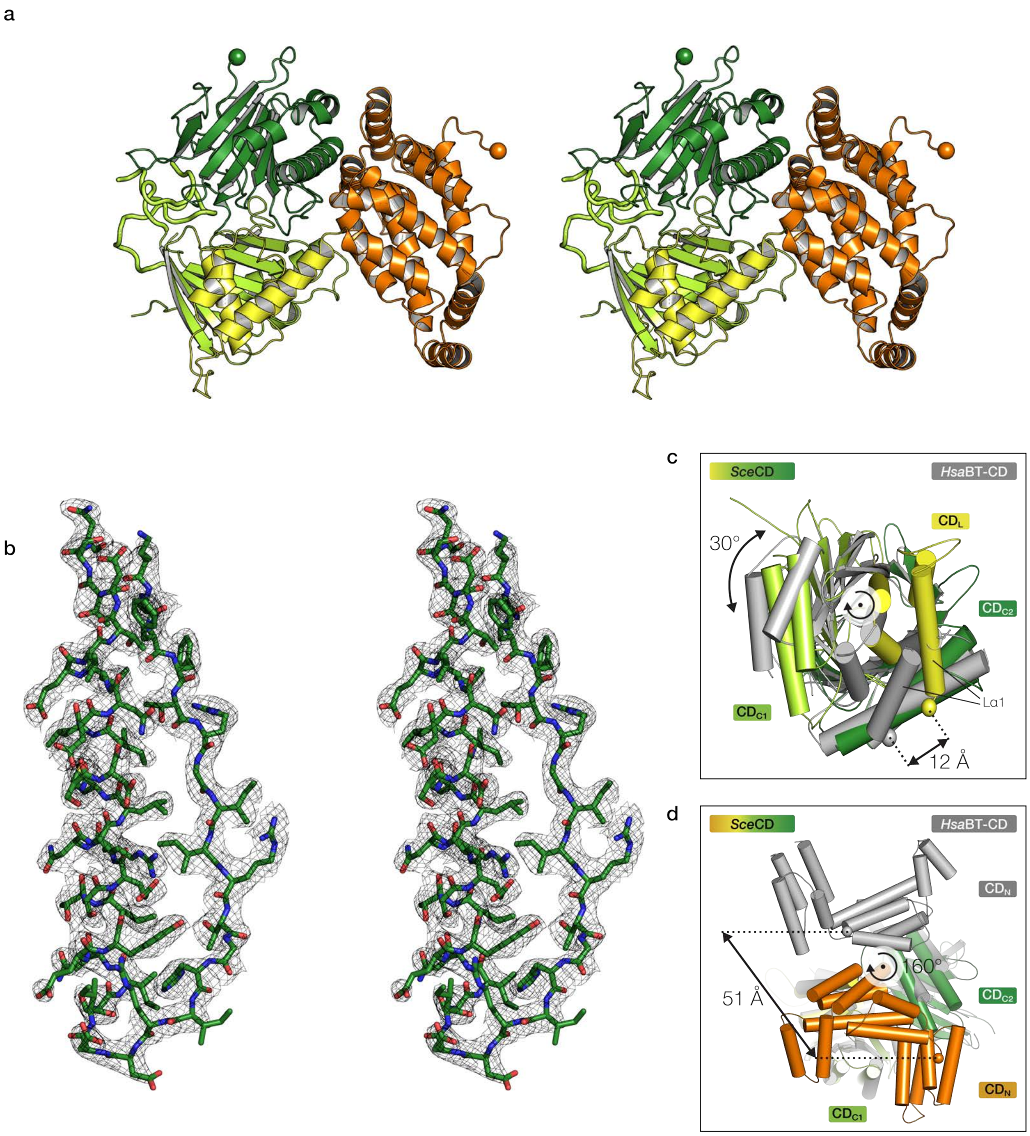

Supplementary Figure 1: Structure of *Sce*CD and comparison to *Hsa*BT-CD. (a) Stereo view of *Sce*CD accompanying Fig 1b. **(b)** Stereo view of a representative part of the electron density in CD_{C2} of *Sce*CD. Shown is a 2Fo-Fc map contoured at 1 σ. **(c)** 30° rotation of CD_{C1} between structures of *Sce*CD and *Hsa*BT-CD visualized based on CD_{C2} superposition. *Sce*CD is shown in color and *Hsa*BT-CD is shown in gray. Both CD_N domains and the *Hsa*BT domain were omitted for clarity. The α-helix Lα1 of both structures, the domain rotation and the distance between N-termini (shown as spheres) is indicated. **(d)** A 160° rotation relates CD_N in *Sce*CD to *Hsa*BT-CD based on a superposition of CD_L/CD_{C1}. *Hsa*BT domain was removed for clarity. Spheres indicate the N-termini and the coloring is as in (c).

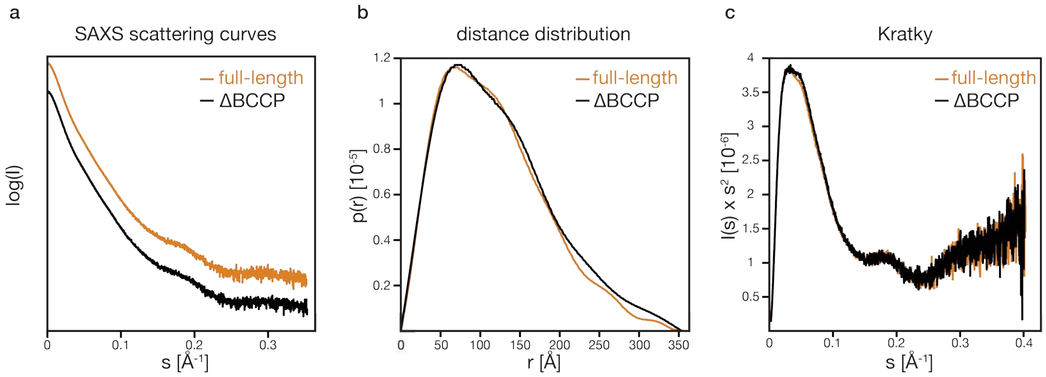

Supplementary Figure 2: SAXS analysis of fungal ACC. Scattering curves **(a)**, distance distribution **(b)** and Kratky plots **(c)** of full-length *Cth*ACC (orange) and *Cth*ΔBCCP (black) demonstrating that BCCP-deletion has no impact on the overall solution structure. For clarity, scattering curves are shown with a y-axis offset.

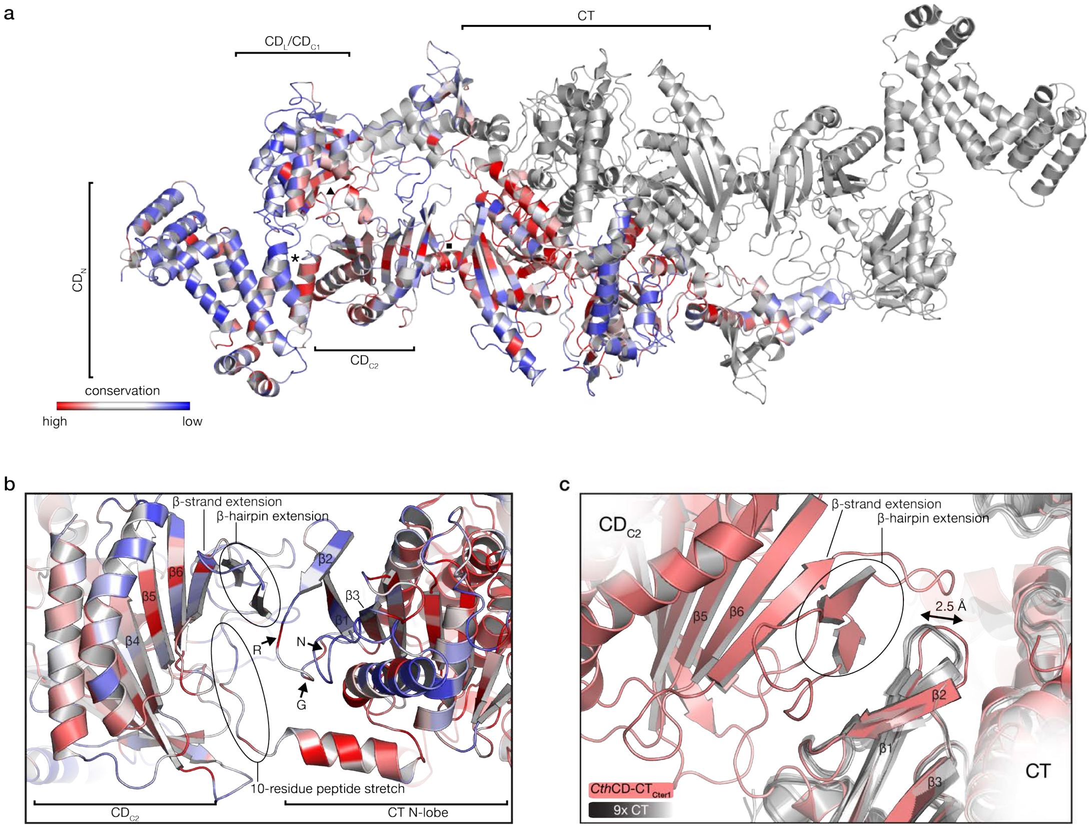

Supplementary Figure 3: Conservation analysis of *Cth*ACC. (a) Cartoon representation of *Cth*CD-CT with one protomer colored according to conservation in a gradient from high (red) to low (blue). The second protomer is colored in gray. Contact regions of CD_N and CD_L/CD_{C1} (asterisk), CD_{C1} and CD_{C2} (triangle) and CD_{C2} and CT (square) are surrounded by regions of higher conservation. **(b)** Close-up on the edge-like hinge between CD_{C2} and CT. Highly conserved residues of the RxxGxN motif are indicated as well as important strands of CD_{C2} and CT. The second CT protomer is omitted for clarity. **(c)** Superposition of nine isolated CT crystal structures (gray) onto the CT domain of *Cth*CD-CT_{Cter1} (red). The loop preceding the conserved RxxGxN motif is displaced in the CD-CT contact by 2.5 Å compared to isolated crystal structures. PDB accession codes of structures used for superposition: 1od2, 1uys, 1uyt, 1w2x, 3h0j, 3h0q, 3k8x, 3pgq, 3tv5.

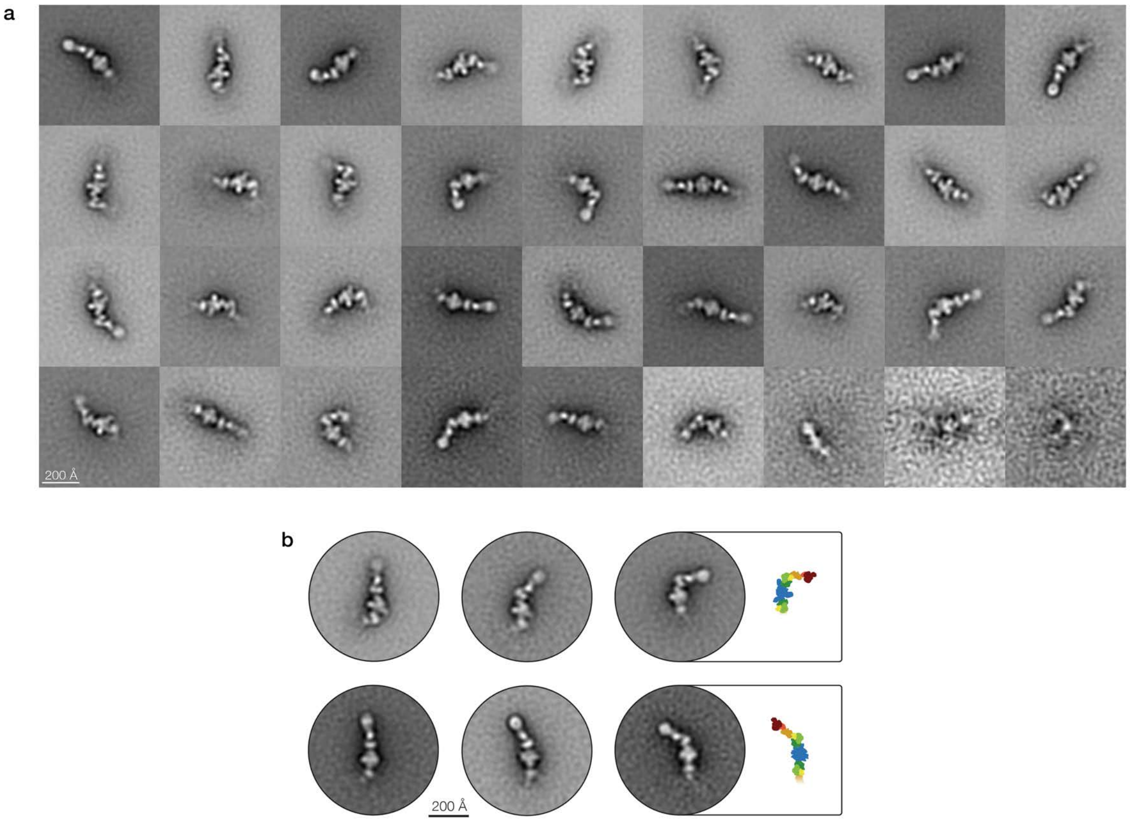

Supplementary Figure 4: Negative stain electron microscopy class averages of *Cth*ACC illustrating its flexibility. (a) 18031 particles were 2D-classified into 36 classes using the MLF2D method. Classes are sorted by number of particles per class, with the highest number at the top left and the lowest number at the bottom right. Scale bar 200 Å. **(b)** 2D-class averages from negative stain EM analysis depict the ensemble of solution conformations of the intrinsic flexibility of *Cth*ACC. Scale bar 200 Å.

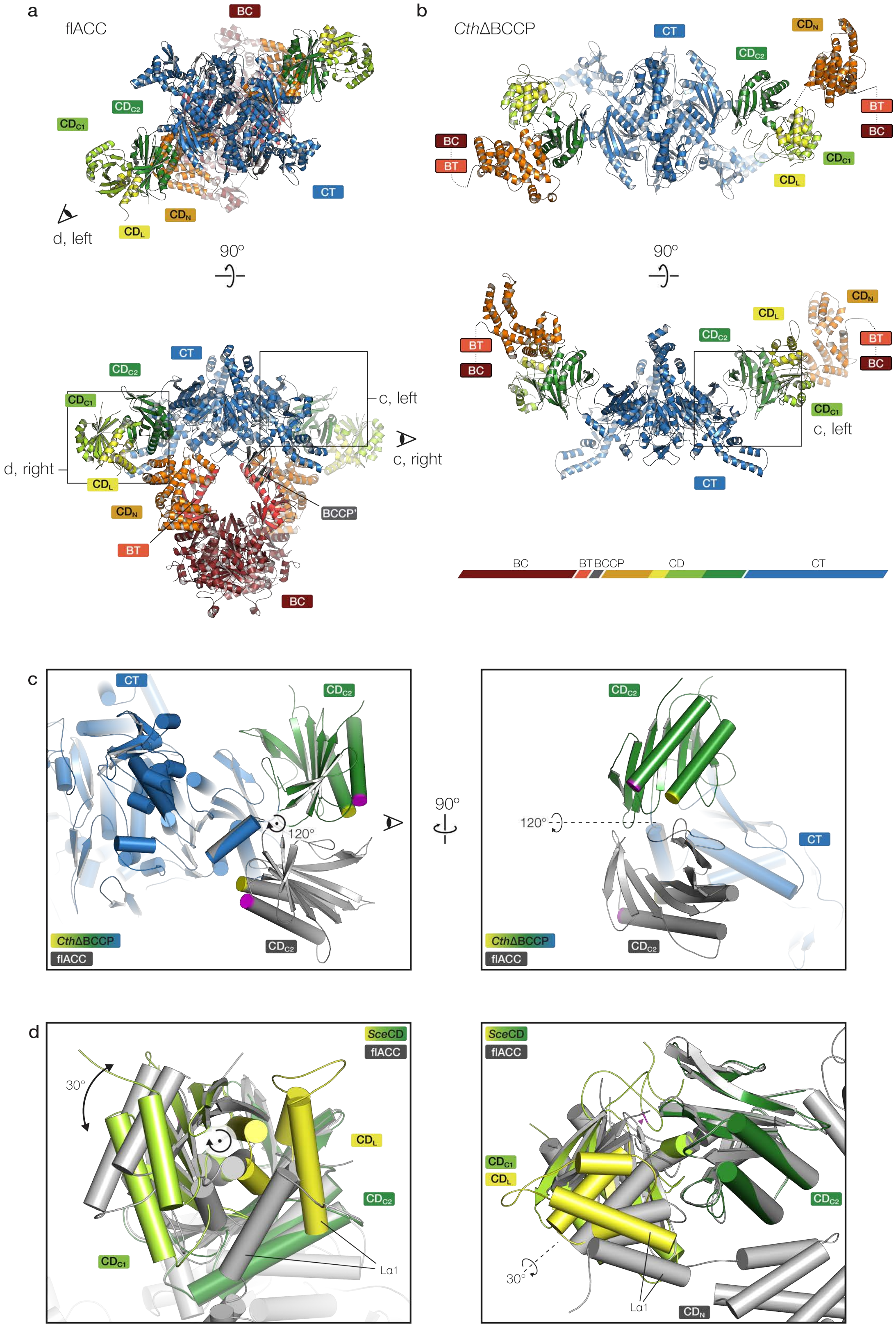
a
flACC
BC
CD_{C2}
CD_{C1}
CT
CD_N
CD_L
d, left
90°
CT
CD_{C2}
CD_{C1}
c, left
c, right
d, right
CD_L
CD_N
BCCP'
BT
BC
b
*Cth*ΔBCCP
CT
CD_N
CD_{C2}
BT
BC
BC
BT
CD_{C1}
CD_L
90°
CD_L
CD_N
CD_{C2}
BT
BC
BT
BC
CD_{C1}
c, left
CT
BC
BT
BCCP
CD
CT
c
CT
CD_{C2}
120°
*Cth*ΔBCCP
flACC
CD_{C2}
90°
CD_{C2}
120°
CT
*Cth*ΔBCCP
flACC
CD_{C2}
d
*Sce*CD
flACC
30°
CD_L
CD_{C2}
CD_{C1}
Lα1
*Sce*CD
flACC
CD_{C1}
CD_L
CD_{C2}
30°
Lα1
CD_N

Supplementary Figure 5: Conformational variability in fungal ACC. (a) Cartoon representation of triangular flACC (PDB accession code: 5csl). In (a) and (b) domains are colored according to the linear sequence scheme in (b). **(b)** Cartoon representation of *Cth*ΔBCCP. The orientations of (a) and (b) are based on a superposition of the CT domains. Fields of view for panels (c) and (d) are indicated with boxes and eyes. **(c)** Left: Detailed view of the CD_{C2}/CT 120° hinge motion viewed along the rotation axis. *Cth*ΔBCCP is colored and flACC is shown in gray for clarity. Corresponding ends of CD_{C2}-helices are colored in yellow and magenta. The rotation axis is indicated and the view for the right subpanel is indicated with an eye. Right: View onto the rotation axis. **(d)** Conformational states of the CD_{C1}-CD_{C2} hinge in the non-phosphorylated flACC and in the phosphorylated *Sce*CD crystal structures when superimposed on CD_{C2} domains. Lα1, the first N-terminal helix of the CD_L domain, is labeled to illustrate the conformational differences. The left panel shows the view along the rotation axis, the right panel corresponds to a view onto the axis. The position of the phosphorylated Ser1157 in the regulatory loop is indicated by a magenta triangle.

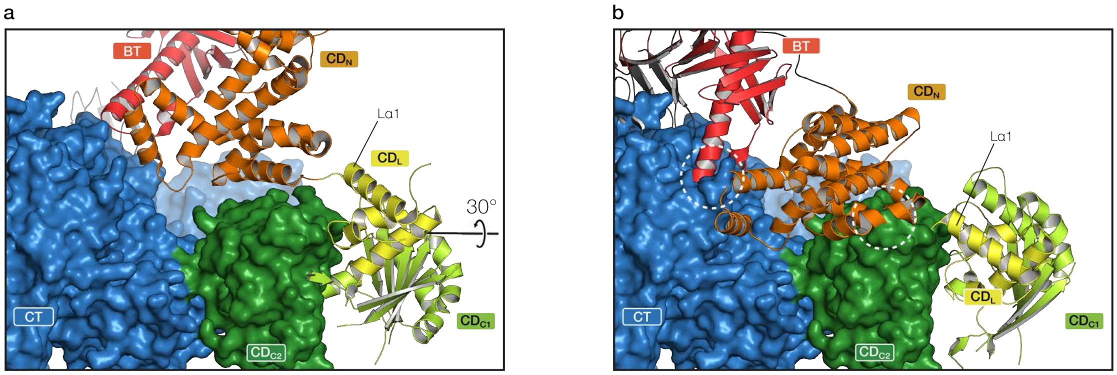

Supplementary Figure 6: Conformation of phosphorylated CD_{C1}-CD_{C2} hinge is incompatible with BT/CD_N conformation in flACC. (a) Detailed view on flACC (PDB accession code: 5csl) with CT and CD_{C2} domains shown as surface and BT, BCCP, CD_N, CD_L and CD_{C1} domains shown as cartoon **(b)** flACC model with the CD_{C1}-CD_{C2} hinge conformation as observed in the *Sce*CD crystal structure. Clashes of BT/CD_N with CT and CD_N with CD_{C2}, that are caused by the 30° rotation of CD_{C1} relative to CD_{C2} (rotation axis shown in (a)), are indicated with white circles. For orientation, the first helix of CD_L is labeled in both panels.

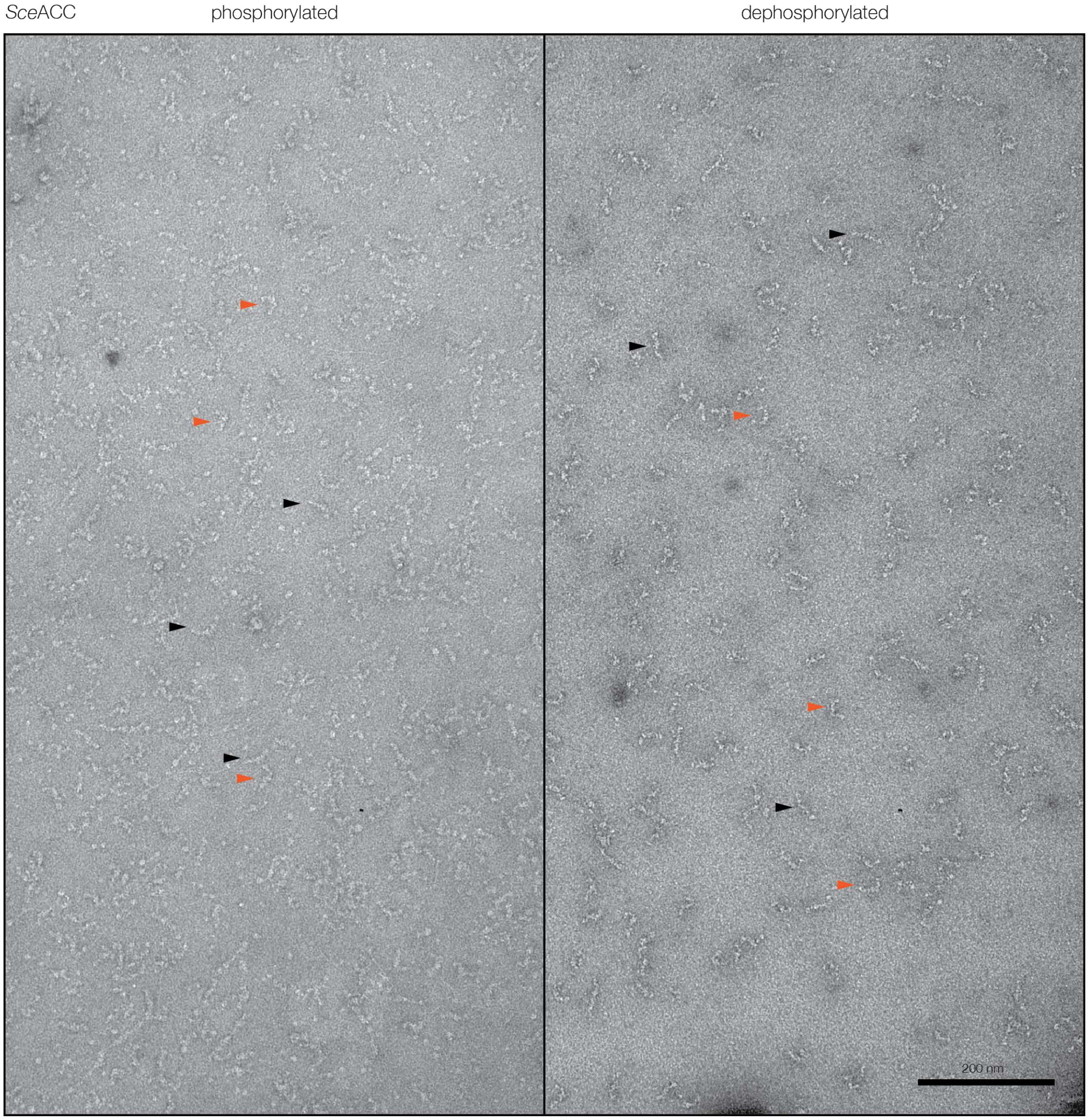

Supplementary Figure 7: Negative stain electron microscopy of phosphorylated and dephosphorylated *Sce*ACC shows similar degree of conformational flexibility. Micrographs of phosphorylated (left) and dephosphorylated *Sce*ACC (right) are shown side by side. Selected particles representing elongated and U-shaped molecules are indicated with black and orange arrows, respectively. The scale bar is 200 nm.

Supplementary Table 1: Small angle x-ray scattering data collection and processing

	full-length *Cth*ACC	*Cth*ΔBCCP
Instrument	B21 at Diamond Light Source	B21 at Diamond Light Source
Beam size (mm)	1 x 5	1 x 5
Wavelength (Å)	1	1
q range ($Å^{-1}$)	0.005 - 0.4	0.005 - 0.4
Detector distance (m)	3.92	3.92
Temperature (K)	293	293
Protein concentration (mg/ml)	2.5, 5, 10	2.5, 5, 10
Scan repeats	30	30
Total exposure time (s)	300	300
Capillary diameter (mm)	1.6	1.6
I(0) ($Å^{-1}$) [from P(r)]	0.023	0.024
Rg (Å) [from P(r)]	94	97
Rg (Å) (from Guinier)	89 ± 2	92 ± 3
Porod volume estimate ($Å^3$)	1176290	1174390
Dmax (Å)	350	350
